# Trauma stabilization points in armed conflict: operational evolution and strategic reflections from mosul to Gaza

**DOI:** 10.1007/s00068-026-03260-y

**Published:** 2026-07-24

**Authors:** Caviglia Marta, Pigozzi Luca, Hsiao Kai-Hsun, Abdel-Fattah Areej, Gargavanis Athanasios, El Ghoul Wessam, Zimmerman Jonas, von Schreeb Johan, Ragazzoni Luca, Salio Flavio

**Affiliations:** 1 CRIMEDIM - Center for Research and Training in Disaster Medicine, Humanitarian Aid and Global Health, Università del Piemonte Orientale, via Lanino 1, 28100 Novara, Italy; 2 Department of Translational Medicine, Università del Piemonte Orientale, Novara, Italy; 3 World Health Organization Office for Occupied Palestinian territory, Gaza, Palestinian Territory Palestinian Territory; 4 Department of Medicine, University of Oviedo, Oviedo, Spain; 5 Research Group on Prehospital Care and Disasters - GIAPREDE, Health Research Institute of the Principality of Asturias, Oviedo, Spain; 6 World Health Organization, Geneva, Switzerland; 7 Department for Sustainable Development and Ecological Transition, Università del Piemonte Orientale, Vercelli, Italy; 8 World Health Organization Regional Office for the Eastern Mediterranean, Cairo, Egypt; 9 Department of Global Public Health, Karolinska Institutet, Stockholm, Sweden; 10 Emergency Department, Karolinska University Hospital, Stockholm, Sweden; 11 Institute of Clinical Sciences, Sahlgrenska Academy, University of Gothenburg, Gothenburg, Sweden; 12 Center for Disaster Medicine, University of Gothenburg, Gothenburg, Sweden

## Abstract

**Background:**

Forward trauma care remains a critical gap in emergency responses to armed conflict, particularly where conventional medical evacuation is disrupted. Trauma Stabilization Points (TSPs) were introduced in previous conflicts to bring life-saving care closer to the point of injury. To improve standards and adaptability, the World Health Organization convened a Technical Working Group to develop operational guidance for TSPs, drawing on diverse field experiences, including the ongoing Gaza deployments.

**Case presentation:**

Between February and July 2024, three TSPs were established in Gaza, managing over 4,000 consultations. Initially focused on trauma stabilization and referral, these sites quickly adapted to minor injuries and non-traumatic conditions, reflecting population needs and access challenges. Referral rates varied across sites due to hospital proximity, ambulance availability, and shifting frontlines. Security threats limited forward deployment and safe patient access, requiring high mobility and rapid relocation. Experience from Gaza highlighted key operational principles: locating TSPs near the point of injury; integrating within a functioning trauma referral pathway supported by evacuation capacity and hospital readiness; maintaining clear clinical functions and staffing standards; and using standardized documentation for quality assurance and continuity. Lessons from Gaza aligned with those from other conflict zones, emphasizing challenges such as insecurity, fragmented oversight, and disrupted referral systems. The guidance recognizes the need for adaptable models that balance mobility with advanced interventions, including damage control resuscitation in austere settings.

**Conclusions:**

The Gaza experience, together with lessons from other conflict settings, is shaping the development of flexible, context-sensitive operational guidance for TSPs. This guidance aims to support emergency care actors and national authorities in determining when and how to deploy TSPs in complex emergencies, balancing core trauma functions with the realities of modern warfare.

## Background

Modern conflicts increasingly involve asymmetric warfare, urban combat, and deliberate or incidental targeting of health infrastructure, significantly complicating timely trauma care [1–3]. Contemporary military trauma systems, structured from the point of injury (POI) to definitive treatment across successive echelons, have improved survival and functional outcomes of wounded soldiers, [4–7] and informed civilian forward-focused models such as Trauma Stabilization Points (TSPs), which may be conceptualized as civilian equivalents of forward military medical capabilities such as NATO Role 1 facilities, though operating under fundamentally different mandates, resource realities, and governance frameworks. The use of TSPs in emergency response settings as a structured component of trauma response emerged during the 2016–2017 battle for the recapture of Mosul, Iraq, then under the control of the Islamic State of Iraq and Syria [8,9]. The World Health Organization (WHO), in collaboration with the local health authorities, implemented a three-tier trauma care and referral system, adapting the military continuum of care model to meet the needs of civilian populations [8–10] At its core, TSPs were mobile units located 10 to 15 min from the frontlines, capable of relocating as conflict lines shifted [8,9]. Managed by non-governmental organizations (NGOs), these mobile units aimed to receive trauma cases earlier near the POI, provide rapid clinical stabilization and triage, optimize the use of limited transportation resources, and reduce the burden on receiving hospitals by ensuring that patients requiring advanced treatment were transferred to higher levels of care, typically field hospitals positioned approximately one hour away [8,9]. In seeking to establish a structured and regulated trauma pathway, WHO expanded the array of involved actors to include Emergency Medical Teams (EMTs). EMTs typically deploy emergency care in sudden-onset disaster settings and have set standards and levels of care [11]. Under this framework, the TSPs were functionally aligned as a special form of Type 1 Mobile EMT and consistently focused on prehospital trauma care, while the subsequent field hospitals correspond to Type 2 EMTs [11]. This alignment promoted adherence to minimum standards, including clinical capabilities, quality of care, documentation, self-sufficiency, and operational neutrality [12].

Despite operational challenges [11], including delayed evacuations and surgical interventions, fragmented coordination between civil and military actors, limited integration with post-operative and rehabilitative care, security constraints, and concerns around embedding health workers with military forces, the model demonstrated perceived efficacy and was adapted in other conflict-affected settings, including the Syrian Arab Republic, Ukraine, and Gaza, where TSPs supported care efforts during the 2018–2019 mass demonstrations and continue to do so in the current conflict escalations [13–17] Recognizing TSPs’ potential and recurring challenges, the WHO EMT secretariat convened a Technical Working Group (TWG) of experts and practitioners with direct experience in TSP implementation across humanitarian and, to a lesser extent, military contexts. The TWG aimed to develop flexible, context-sensitive guidance reflecting operational realities rather than rigid standards. Accordingly, the objective of this manuscript is threefold: (1) to trace the conceptual and operational evolution of TSPs and their integration within the trauma care pathway, as an empirical contribution intended to inform the ongoing development of a WHO TSP operational reference guide; (2) to identify the gaps between theoretical frameworks and operational realities, with a focus on the current emergency response in Gaza; and (3) to provide critical reflections for optimizing future deployments. By combining this descriptive analysis of operational data from Gaza TSPs with a comparative, concept-driven evaluation of prior and current TSP implementations in other conflict settings, this manuscript identifies key operational determinants of TSP effectiveness and derives generalizable lessons to inform future deployment and the development of WHO guidance. In doing so, it contributes to the ongoing discourse on delivering timely and effective trauma care in volatile, resource-constrained settings marked by asymmetric warfare, shifting frontlines, and attacks on health care. While this manuscript does not present WHO guidance itself, it provides evidence base grounded in field implementation experience to inform the ongoing development of such guidance.

## Case presentation

### Towards a WHO TSP guidance document: operational principles and lessons from the field

This section outlines the operational principles and conceptual framework underpinning TSP deployment, which provide the basis for interpreting field observations. The WHO TSP guidance currently under development by the TWG is intended as a practical resource for national and local authorities and emergency responders operating in sudden-onset disasters and conflict settings. Drawing on prior consensus-based studies [1] and operational field experience from TSP implementation, the TWG sought to develop adaptable recommendations grounded in field realities, centred on the principle that TSPs should be deployed when the health systems cannot cope with the trauma caseload surges, whether due to the emergency scale or inherent limitations of the health infrastructure and services. The aim of this evolving guidance is to provide clarity on key operational components essential for fulfilling the TSP role along a structured trauma care continuum, including (a) appropriate positioning near the POI; (b) integration within a functional referral and trauma pathway; (c) definition of clinical functions and minimum staffing configurations; and (d) the use of standardized documentation and data collection tools.

Effective TSP deployment requires proximity to the frontline for rapid interception of severe trauma cases near the POI, enabling early life-saving and limb-saving care and resuscitation to mitigate the impact of distance and geographic challenges on evacuation times, before transferring patients to surgical-capable facilities. While military doctrine set a 10-minute response window, the civilian adaptation accepts up to 20 min, an adjustment reflecting the practical constraints faced by emergency responders in accessing the scene due to insecurity or limited coordination with combatant forces [18,19] This interval may be covered by ambulances or, for shorter distances, on foot or stretcher. It is worth mentioning that early assessments from the Mosul operations attributed the TSP model with saving an estimated 1,500 to 1,800 lives, due to positioning close to the POI and shortening evacuation to surgical-capable facilities [9] However, frontline placement raises significant operational challenges. TSPs must maintain high mobility in response to shifting frontlines or deteriorating security conditions, limiting the range of medical capabilities and resources they carry. This concern is amplified in an era where, despite the protections ostensibly provided by International Humanitarian Law (IHL), healthcare workers, infrastructure, and vehicles are increasingly targeted [20] Field reports from Mosul underscored this vulnerability, highlighting the heightened risks to both humanitarian personnel and patients due to the proximity of TSPs to active conflict zones [11] Additionally, the increasing use of drones and aerial surveillance in contemporary conflicts significantly constrains the feasibility of forward medical deployment. ^21^ Unlike previous conflicts, where proximity to the POI was more readily achievable, drone-enabled targeting reduces concealment, increases risk to healthcare workers, and limits mobility. This has been particularly evident in Ukraine, where forward medical units are frequently relocated, concealed, or placed in hardened structures. These dynamics challenge the core assumption underpinning the TSP model namely, safe proximity to the POI and underscore the need to adapt strategies, including modularity and enhanced protective measures in ways that preserve access to civilians particularly in densely populated areas where needs are greatest despite heightened operational risks [21].

The TWG emphasized that TSPs must integrate within a structured trauma pathway, which requires medical evacuation via ambulances or designated vehicles on safe routes, and sufficient receiving hospital capacity. Absence of either disrupts the continuum of care, delaying damage control, surgical treatment, and prolonging patient stays inside facilities ill-equipped for definitive surgical care or prolonged hospitalization. Therefore, TSPs deployment should assume at least the initial fulfilment of these conditions. Nevertheless, conflict settings are inherently dynamic and unpredictable, and even initially adequate conditions may deteriorate. For example, during Mosul, secondary-level surgical capacity was compromised at times due to alternative referral pathways and sudden patient surges.

In fulfilling their primary functions, TSPs are mandated to perform triage, initially distinguishing between walking and non-walking casualties, and subsequently prioritizing those with life- or limb-threatening injuries. Accordingly, core interventions encompass haemorrhage control, acute airway management, stabilization of major long-bone and pelvic fractures, pain management, and initial damage control resuscitation. Lessons from past deployments, where some NGOs expanded the initially basic WHO EMT guidance to match their medical teams’ advanced capabilities [9], highlighted the potential for variability in scope based on staff expertise. The Ukraine experience was particularly influential on this matter, as large-scale combat operations challenged evacuation timelines, and required advanced resuscitation near the frontline [22]. Whole blood transfusions, including low-titer O whole blood and “walking blood banks,” were implemented in austere conditions with unreliable refrigeration, electricity, and evacuation systems, defining the scope and scalability of interventions [22] The TWG recognized that while not all TSPs can provide transfusion or advanced resuscitation, modern warfare increasingly demands adaptable models balancing agility with higher-level care, accounting for operational constraints. Advanced resuscitation in Ukraine also revealed challenges with clinical governance and maintaining medical standards, with reported absent centralized oversight, inconsistent supervision, and fragmented command [22] Operational barriers of prehospital transfusion (including prolonged transport times, resource scarcity, variable adherence to guidelines, and decentralized supply management), further demonstrated that outcomes are highly context-dependent [23] Thus, while standardization and centralization are desirable, they are often unfeasible in contemporary conflict settings, prompting the TWG to define core competencies and procedures while allowing scalable, context-specific life-saving interventions.

Staffing variations influence care quality and referrals. During the 2018–2019 Gaza demonstrations, Ministry of Health (MoH)-led TSPs staffed by physicians and nurses contrasted with PRCS TSPs staffed by paramedics and EMTs, resulting in higher hospital referrals from the latter due to limited on-site treatment capabilities [16] Core trauma staff should include medical doctors, nurses, and trained paramedics, working cohesively under established protocols with rotating shifts for 24/7 coverage [12] Beyond professional categories, effective deployment requires structured pre-deployment preparation and ongoing training in tactical and disaster medicine, including mass casualty management, damage control resuscitation principles, and standardized tactical care competencies, consistent with established frameworks and other proposed training models, adapted for civilian context [24].

While rapid stabilization and referral of major trauma remain central, a dual-use function emerged already during the Mosul operations when, amid the large-scale displacement of the civilian population, TSPs increasingly delivered definitive care for minor trauma and non-traumatic emergencies to relieve secondary facilities. This capacity has since become integral to the TSP mandate [25] For instance, during the 2018–2019 mass demonstration in Gaza, TSPs managed approximately 4,255 cases over two months, primarily involving tear gas inhalation, of which 54% were treated and discharged on-site [16] This observation, which reflects operational adaptation to population needs and health system constraints rather than a formal or intended expansion of the TSP clinical mandate, has led the TWG to consider how best to balance a trauma-focused approach with the practical realities of caring for non-trauma cases, while also exploring a potential role in providing elements of prolonged field care when rapid transfer is not feasible.

Documentation is critical for operational decision-making, performance evaluation, and continuous improvement [4] Accordingly, the TWG underscored the importance of systematic patient registration and intervention recording, recommending the use of locally available tools while outlining a minimum dataset in a standardized format. This dataset includes key elements such as patient demographics (sex, age), estimated time of injury, mode of transport, mechanism and anatomical location of injuries, time of arrival at the TSP, vital signs, initial and secondary triage categories, interventions performed, patient destination, and time stamps. This approach is intended to support systematic monitoring and continuous improvement of TSPs across successive operational contexts. However, the feasibility of such detailed documentation in austere environments or high-threat situations may be limited.

### The use of TSPs in Gaza: operational challenges and deviation from current frameworks

**Fig. 1:**
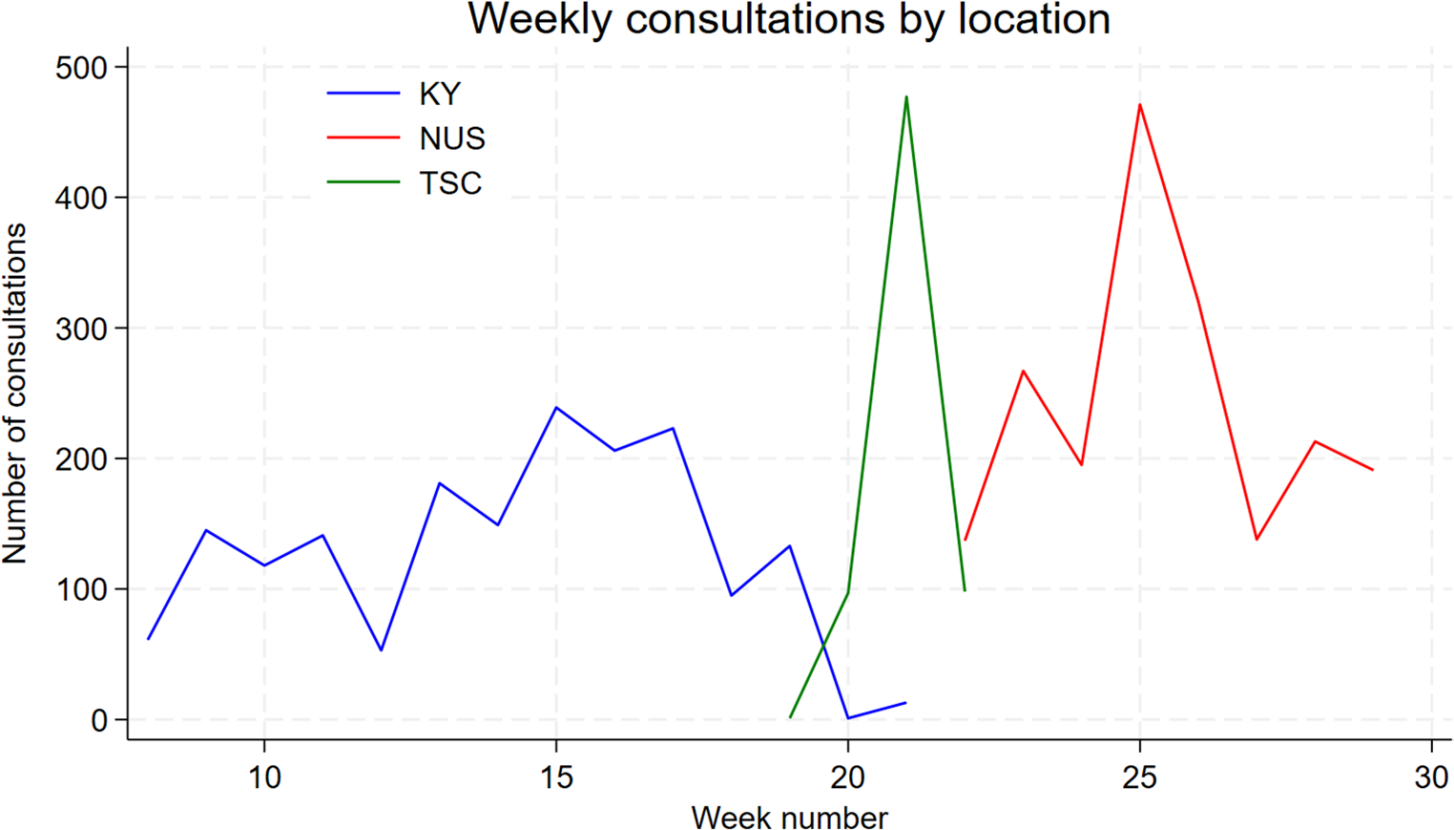
Weekly Consultations at the three Gaza Trauma Stabilization Points (TSPs)

Building on this framework, the Gaza deployment represents the primary empirical basis of this analysis, providing real-world operational data to examine the applicability and limitations of existing TSP frameworks. Although operational for approximately five months, the TSP concept was implemented during the ongoing conflict in Gaza to ensure immediate access to lifesaving surgical care [26] Between February and July 2024, three TSPs were deployed, each supported by three to five PRCS ambulances for referral to secondary care. Collectively, TSPs conducted approximately 4,000 consultations, with surges during certain periods (Fig. 1).

**Fig. 2:**
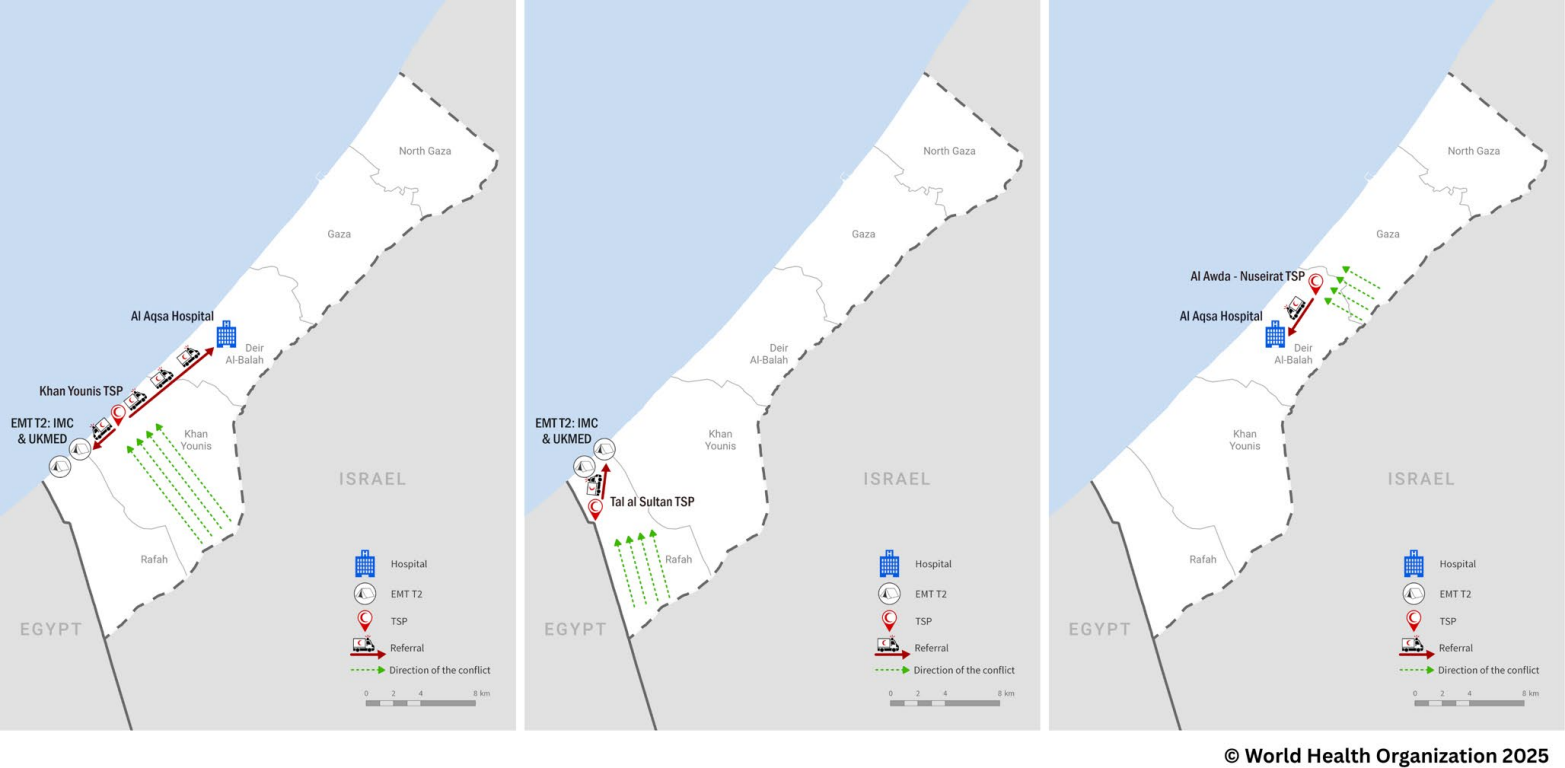
Locations and referral pathways of the three Gaza Trauma Stabilization Points (TSPs) [1] IMC, International Medical Corps ^1^The designations employed and the presentation of the material in this publication do not imply the expression of any opinion whatsoever on the part of WHO concerning the legal status of any country, territory, city or area or of its authorities, or concerning the delimitation of its frontiers or boundaries. Dotted and dashed lines on maps represent approximate border lines for which there may not yet be full agreement

Data were extracted from a WHO-owned dataset and supplemented by operational insights from WHO field teams. Statistical analyses were performed using STATA 19. In February 2024, amid a humanitarian crisis affecting 2.3 million people and resulting in over 29,000 deaths and 69,000 injuries [27], the first TSP was established in Khan Younis by PRCS, with technical support from WHO and the international Cadus EMT (Fig. 2).

This tent-based facility was strategically positioned along th westward evacuation route of civilians fleeing the conflict, inside the so called “al Mawasi humanitarian zone” [28] and on a major road connecting northern and southern Gaza, enabling two referral pathways: north to Al Aqsa Hospital, supported by the MoH and specialized EMTs, and south to EMT Type 2 field hospitals operated by the International Medical Corps (IMC) and UK-Med. Its proximity to the Nasser Medical Complex, a tertiary facility that became non-functional and inaccessible in February 2024, also facilitated recognition by civilians and ambulance networks. Operating until May 2024, the TSP averaged 125.6 consultations per week, with 15% referred and 84% discharged (Table 1).

**Table 1:** Weekly consultations, referral rate and discharge rates from the three Gaza Trauma Stabilization Points (TSPs)

Location	Weekly Consultations (Mean)	Referral Rate (Mean ± SD)	Discharge Rate (Mean ± SD)
Khan Younis	125.6	0.15 ± 0.30	0.84 ± 0.33
Tal Al Sultan	168.3	0.44 ± 0.53	0.56 ± 0.53
Al Nuseirat	241.5	0.17 ± 0.24	0.41 ± 0.51

**Fig. 3:**
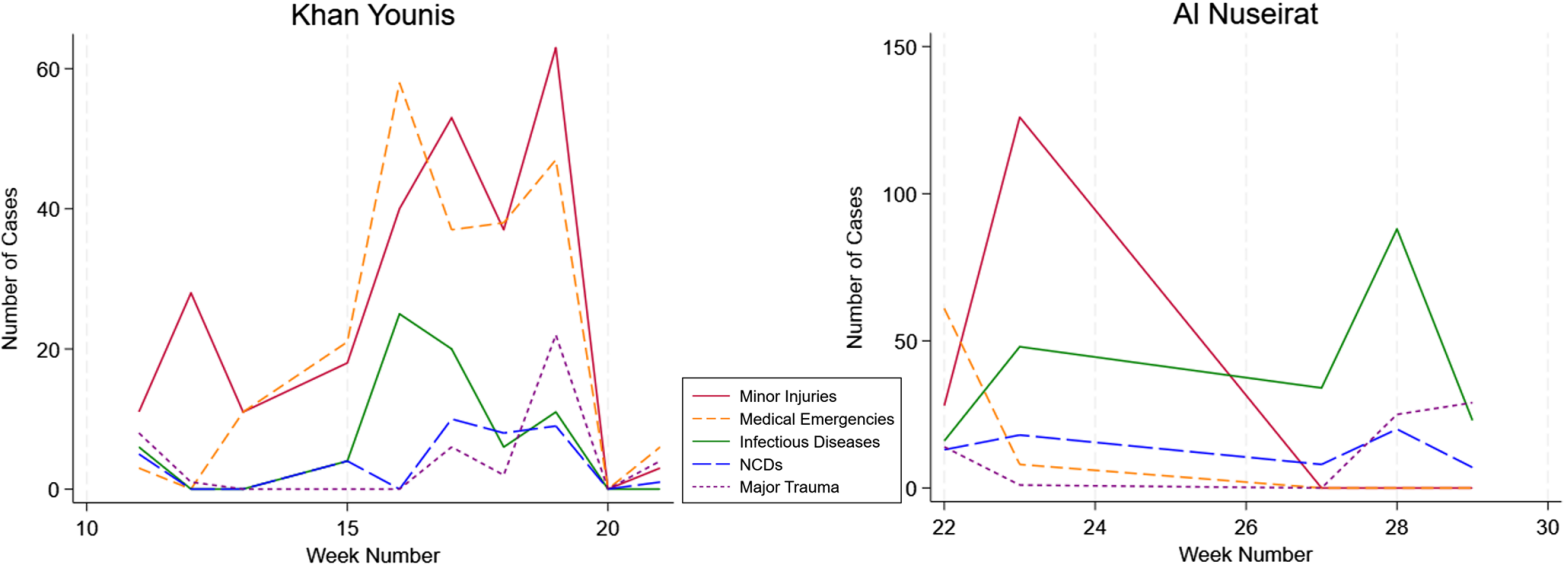
Weekly trends of clinical cases at the Khan Younis and Al Nuseirat Trauma Stabilization Points (TSPs), categorized by type of presentation

**Fig. 4:**
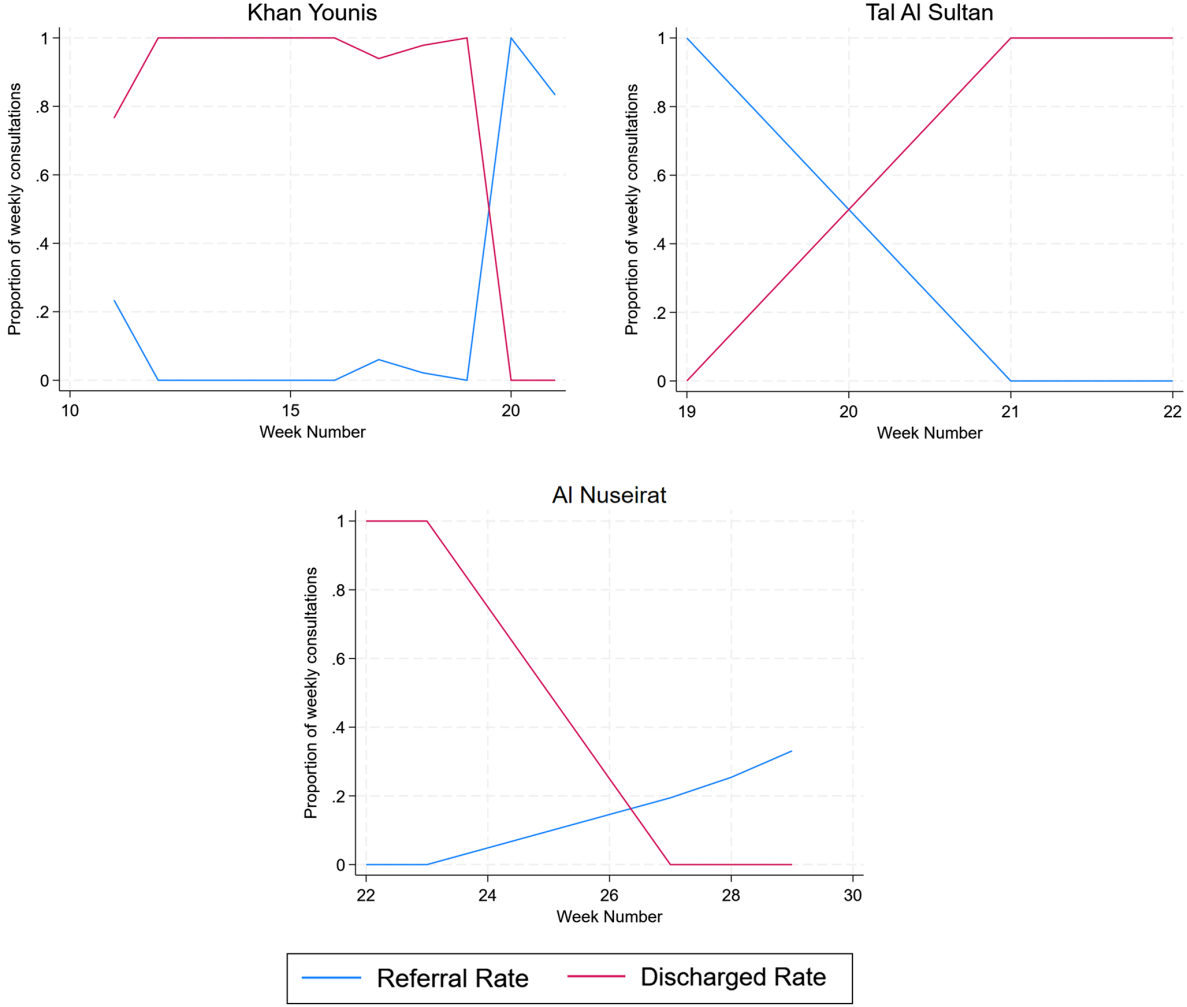
Weekly trends in referrals and discharges from the three Gaza Trauma Stabilization Points (TSPs)

Initially a stabilization and discharge point for minor trauma and emergencies (Fig. 3), the facility gradually expanded to broader primary care services. In the final phase (Weeks 20–21), it transitioned to a referral-only role due to shifting frontline and limited number of cases (Fig. 4).

In May 2024, following the escalation of hostilities around Rafah, Cadus and Médecins Sans Frontières (MSF) Belgium, under WHO guidance and technical leadership, established a second TSP in the pharmaceutical warehouse of an existing primary healthcare centre in Tal al Sultan. Active for about one month, it managed 673 consultations (168.3 per week; Table 1). Clinical category data were incomplete and thus excluded from Fig. 3. Referral pathways were directed toward the UK-Med and IMC EMT Type 2 (Fig. 2). The TSP reported the highest referral rate (44%) and a 56% discharge rate on average, both with very high variability. Weekly trends showing a progressive inversion between the referral rate and the discharged rate after the first week.

Following the takeover of Rafah, WHO supported Cadus in establishing a third TSP in the Middle Area, anticipating evolving conflict dynamics. This TSP was s positioned in front of Al Awda Maternity hospital in Nuseirat, a well-known private gynaecological centre already integrated within the local ambulance referral network (Fig. 2). Operational for about one month, it managed 1,932 patients, (241.5 per week), mostly minor trauma initially, with a late surge in infectious diseases. Referral and discharge rates were 17% and 41%, respectively.

### Strategic reflections for optimizing future deployments

Drawing on the operational framework and the empirical analysis of TSP implementation in Gaza, where field realities often diverged from the theoretical model, this section synthesizes key operational lessons (Table 2). These insights are further informed by comparisons with prior TSP deployments and considered in relation to their implications for future operations and the ongoing development of WHO guidance. The three TSPs deployed in Gaza responded to hospital collapse or inaccessibility amid overwhelming trauma caseloads, consistent with the guidance deployment triggers. However, operational patterns observed revealed deviations from the theoretical model, reflecting context-specific constraints typical of asymmetric warfare and dynamic, resource-limited environments.

One key deviation involved proximity to the POI. For example, while initially the Khan Younis TSP was located along a major road close to the POI, shifting frontlines reduced its proximity over time. The other two TSPs were established within pre-existing infrastructure, limiting both their mobility and proximity to the frontline. None had modular features to allow relocation as the frontline shifted, a factor that should be anticipated during planning in protracted or dynamic conflicts. As the conflict lines evolved, no additional TSPs were deployed because casualties and ambulances could reach secondary facilities directly. At the same time, EMT Type 1 mobile clinics positioned near secondary hospitals (which in principle could have operated as TSPs close to the POI) were repurposed to manage minor cases after triage and stabilization at the secondary level. Similar constraints emerged in Ukraine, where prolonged evacuation times prompted the deployment of TSPs closer to POIs with a broader functional scope, including extended stabilization and surgical interventions, often concealed in basements or improvised shelters. While such proximity improved access, it also increased exposure to hostilities [29], requiring rapid dismantling and relocation in case of imminent targeting or shifting frontlines. Ensuring TSP mobility and modularity demands not only forward planning but also adequate equipment, transport capacity, and logistical support. This consideration is consistent with recent developments in military and emergency medical approaches that emphasize modular and highly deployable capabilities adaptive to dynamic operational environments [30]. Additionally, integrating TSPs into humanitarian notification systems could support safer relocation in contexts of volatile frontlines, insecurity, and restricted access.

Referral systems also faced challenges. Although three to five PRCS ambulances per TSP and designated hospitals were planned, insecurity and road inaccessibility intermittently delayed transfers, undermining the TSPs’ role as rapid stabilization points [31]. The relatively low referral rates at Khan Younis and Al Nuseirat may therefore reflect not only limited clinical need but also constraints in referral capacity, including prolonged transport times, insecurity, poor road conditions, and ambulance shortages. By contrast, the Tal Al Sultan TSP recorded a higher, though not statistically significant (Kruskal–Wallis: χ²(2) = 2.16, *p* = 0.34) referral rate, likely due to its proximity to referral centres. This illustrates the operational trade-off between placing TSPs close to the POI for rapid stabilization and ensuring timely referral to higher-level facilities.

The scope of clinical functions shifted beyond initial plans. At Khan Younis, conflict-driven displacement increased non-trauma presentations, demonstrating the dual-use nature of TSPs as trauma stabilization and primary care points. While this alleviated hospital burden, it challenged staffing, equipment, and spatial capacity, and risked reframing TSPs as general healthcare facilities. Field experience indicates this trend is not isolated. As noted by Salio et al.,^1^ debate continues over whether TSP mandates should formally include non-trauma care, but no consensus has emerged. Given the persistent healthcare gaps in conflict settings, non-trauma cases are likely to continue increasing [32] Ensuring essential non-trauma care alongside trauma interventions is vital for population trust and response effectiveness. Rather than expanding the TSP mandate outright, a pragmatic approach could be to actively monitor case types and periodically reassess whether the TSP model remains suitable or if an adaptation, such as transitioning to a standard EMT Type 1 or co-locating such a facility with a TSP, would better meet local needs.

Security concerns remain paramount. The evacuation of the Tal Al Sultan TSP on 27 May 2024 due to escalating hostilities illustrates the operational fragility of these facilities [33], with each relocation carrying the risk of abandoning critical assets. The Ukrainian experience similarly demonstrates the need for concealment, night movements, and dispersed supply caches. In response, WHO emphasized the need for pre-established evacuation protocols and for TSPs to receive the protections guaranteed to medical facilities under IHL. Enhancing TSP mobility through modular design and pre-positioned supply kits could also improve adaptability to shifting frontlines and reduce security risks. At the same time, reinforcing their legal and symbolic status as protected medical spaces may help maintain continuity of care in volatile environments.

Another critical limitation concerned data collection and documentation. Existing datasets enabled analysis, but their incompleteness and low granularity limited detailed evaluation. Although the EMT minimum dataset is theoretically comprehensive, collecting data during acute emergencies remains difficult, a challenge also reported by EMTs in sudden-onset disasters [34, 35] The dual responsibility placed on medical staff to deliver clinical care and collect data often proves untenable, especially under conditions of high patient volume and limited personnel. Introducing a dedicated data officer within the TSP structure could ensure accurate and consistent documentation without detracting from clinical duties [35] Standardized records would also support aggregation and cross-context comparisons. Metrics such as type of trauma, time to treatment, length of stay, transfer time, and ratio of trauma to non-trauma cases could generate operational insights to guide future deployments. A further limitation was the high number of missing entries, largely from “leaving against medical advice” (LAMA) cases, where patients with minor or non-urgent conditions departed before follow-up. This prompted WHO to add LAMA to the minimum dataset to capture population needs more accurately at the front line.

Of note, healthcare workers in Gaza have been familiar with the value and function of TSPs since 2018 [16]. This prior exposure is crucial for future implementations, as introducing such a concept is considerably easier when there is an existing institutional memory regarding its importance. Notably, ambulance personnel demonstrated rapid adaptation to the TSP model upon its introduction, enabling the effective deployment and operation of these facilities.

**Table 2:** Operational considerations for Trauma Stabilization Point deployment derived from multi context field experience. POI, point of injury; TSPs, trauma stabilization points; EMT, Emergency medical teams; LAMA, leaving against medical advice

Domain	Operational observation	Derived consideration for future deployments
Positioning and proximity to POI	Effectiveness in Mosul and early Gaza linked to proximity to injury sites. In Gaza, shifting frontlines reduced proximity over time	TSPs should be deployed as forward as security allows, but must be designed for dynamic repositioning with predefined relocation triggers and modular infrastructure
Mobility and adaptability	Fixed or infrastructure based TSPs in Gaza lost responsiveness as frontlines shifted	Mobility should be considered a core design requirement, including transport capacity, pre-packed modular kits, and relocation protocols integrated into contingency planning
Integration into referral pathways	Referral effectiveness depended on functioning ambulances and receiving hospitals; Gaza showed intermittent breakdowns due to insecurity	Deployment should only occur when minimum viable referral pathways exist or can be rapidly restored; redundancy in evacuation routes should be planned
Clinical scope of care	Expansion from trauma to non-trauma and primary care in Gaza and Mosul; increased burden but improved population access	TSPs should explicitly anticipate dual use demand, with flexible staffing models and predefined thresholds for transition toward EMT Type 1 or hybrid care points
Advanced interventions capacity	Ukraine demonstrated benefit of advanced resuscitation including whole blood transfusion in prolonged evacuation contexts	Advanced interventions should be context dependent; preauthorization of scalable capabilities (e.g., blood products) should be defined according to evacuation time and logistics capacity
Staffing model	Variation between physician led and paramedic led TSPs influenced referral patterns and treatment capacity	Minimum multidisciplinary staffing should be defined, but role flexibility should be allowed depending on caseload, security and availability of skilled personnel
Security and protection	TSPs were repeatedly exposed to targeting risks and forced relocation (Gaza, Ukraine)	TSPs require explicit integration into humanitarian notification systems and reinforced recognition as protected medical facilities under IHL, with pre planned evacuation procedures
Data collection and monitoring	High clinical workload limited documentation quality; missing data and LAMA cases reduced analytical value	Dedicated data officer or simplified digital tools should be integrated as essential operational component rather than optional function
Dual use and population demand	High proportion of non-trauma cases in Gaza (e.g. tear gas, infectious disease)	TSPs should include structured triage for non-trauma conditions and periodic reassessment of case mix to prevent mission drift or misclassification of facility type
System integration and scalability	Fragmented coordination reduced efficiency in several deployments	TSPs should be embedded in a clearly defined trauma system architecture with explicit escalation pathways toward EMT Type 2 or surgical facilities

## Conclusions

This manuscript combines the analysis of original operational data from the Gaza TSP deployment with a broader, guidance-oriented interpretation aimed at informing the development of WHO operational recommendations. In sum, while the TSP model has demonstrated a degree of adaptability that is vital in complex emergencies, its application in Gaza and other contexts reveals both strengths and vulnerabilities. Operational deviations from the theoretical model, particularly concerning mobility, integration into referral pathways, and scope of care, reflect broader challenges inherent in protracted, high-intensity conflicts, including evolving warfare, changing injury patterns, and increasing attacks on healthcare. A strategic reassessment of how TSPs are designed, deployed, and monitored is essential to ensure that they remain fit for purpose while responding to evolving healthcare needs in volatile humanitarian settings.

## Data Availability

No datasets were generated or analysed during the current study.

## Abbreviations

POI: point of injury
TSPs: Trauma stabilization points
WHO: World Health Organization
NGOs: non-governmental organizations
EMTs: Emergency medical teams
TWG: Technical working group
IHL: International Humanitarian Law
MoH: Ministry of Health
PRCS: Palestinian Red Crescent Society
IMC: International Medical Corps
MSF: Medicine Sans Frontieres
LAMA: Leaving against medical advice

## Declarations

### Patient and public involvement

Patients and/or the public were not involved in the design, or conduct, or reporting, or dissemination plans of this research.
